# Altered gut microbiota–host bile acid metabolism in IBS-D patients with liver depression and spleen deficiency pattern

**DOI:** 10.1186/s13020-023-00795-9

**Published:** 2023-07-19

**Authors:** Liqing Du, Zhaozhou Zhang, Lixiang Zhai, Shujun Xu, Wei Yang, Chunhua Huang, Chengyuan Lin, Linda L. D. Zhong, Zhaoxiang Bian, Ling Zhao

**Affiliations:** 1 School of Integrative Medicine, Shanghai University of Traditional Chinese Medicine, 1200 Cailun Road, Shanghai, China; 2 School of Chinese Medicine, Hong Kong Baptist University, Hong Kong, SAR China; 3 School of Biological Sciences, Nanyang Technological University, Singapore, Singapore; 4 Institute of Brain and Gut Research, Chinese Medicine Clinical Study Center, School of Chinese Medicine, 7 Hong Kong Baptist University Road, Kowloon, Hong Kong, SAR China

**Keywords:** Diarrhea-predominant irritable bowel syndrome, Traditional Chinese Medicine, Liver depression and spleen deficiency, Gut microbiota, Bile acids

## Abstract

**Background:**

Dysregulation of gut microbiota–host bile acid (BA) co-metabolism is a critical pathogenic factor of diarrhea-predominant irritable bowel syndrome (IBS-D). Traditional Chinese Medicine (TCM), instructed by pattern differentiation, is effective in treating IBS-D, in which liver depression and spleen deficiency (LDSD) is the most prevalent pattern. Still, it is unclear the linkage between the LDSD pattern and the BA metabolic phenotype.

**Purpose:**

This study aimed to uncover the biological basis of the LDSD pattern from the BA metabolic perspective.

**Methods:**

Patients with IBS-D completed questionnaires regarding the irritable bowel severity scoring system (IBS-SSS), stool frequency, Stool Bristol scale, and Self-Rating Scales of mental health. Fasting blood and morning feces were collected to analyze the gut metagenome and BA-related indices/metabolites.

**Results:**

IBS-D patients with LDSD had a higher incidence of BA overexcretion (41% vs. 23% non-LDSD) with significant elevations in fecal total BAs and serum BA precursor 7α-hydroxy-4-cholesten-3-one levels. Compared to controls or non-LDSD patients, LDSD patients had a featured fecal BA profile, with higher proportions of deoxycholic acid (DCA), 7-ketodeoxycholic acid, and lithocholic acid. It is consistent with the BA-metabolizing genomic changes in the LDSD gut microbiota characterized by overabundances of 7-dehydroxylating bacteria and BA-inducible genes (*baiCD/E/H*). The score of bowel symptoms (stool frequency and abdominal pain) showing greater severity in the LDSD pattern were positively correlated with *bai*-expressing bacterial abundances and fecal DCA levels separately.

**Conclusion:**

We clarified a differed BA metabolic phenotype in IBS patients with LDSD, which closely correlates with the severity of bowel symptoms. It demonstrates that gut microbiota and host co-metabolism of BAs would provide crucial insight into the biology of the LDSD pattern and its internal relationship with IBS progression.

**Supplementary Information:**

The online version contains supplementary material available at 10.1186/s13020-023-00795-9.

## Introduction

Irritable bowel syndrome (IBS) characterized by abdominal pain and altered stool habits is one of the functional gastrointestinal disorders (FGIDs), affecting 11% of the global population and 6% of the Chinese population [1]. IBS is grouped into four subtypes, including diarrhea-predominant IBS (IBS-D), constipation-predominant IBS (IBS-C), mixed IBS (IBS-M), and un-subtyped IBS (IBS-U), among which IBS-D is more prevalent in China [2]. As a result of complex etiologies and incompletely understood pathogeneses, mainstream medications are symptom-focused and generally have unsatisfactory efficacy [3, 4]. There is an increasing demand for alternative medicine in treating IBS.

Herbal medicine is a frequently chosen alternative approach for the Chinese population. Cohort studies have reported that herbal formulas instructed by Traditional Chinese Medicine (TCM) pattern differentiation are substantially effective in relieving global bowel symptoms of IBS-D, diarrhea, and abdominal pain/distention [5–7]. Liver Depression and Spleen Deficiency (LDSD) is the most general TCM pattern in IBS-D; herbal formulas with liver-soothing and spleen-strengthening properties (e.g., Tong-Xie-Yao-Fang and Xiao-Yao-San) are widely applied to treat IBS-D [8, 9]. Nevertheless, the biological foundation of the LDSD pattern is yet to be clarified, which restricts the development of pharmacology and clinical application of liver-soothing and spleen-strengthening herbal medication.

IBS-D can be triggered by multiple external and internal factors, which include an excess of irritants, e.g., bile acids (BAs), exposure in the colon [10, 11]. BA overexcretion was reported to affect around 30% of IBS-D patients, who exhibit more severe bowel dysfunction, poorer quality of life, and a higher depression score than those with normal BA excretion [12]. Excessive BA excretion in IBS-D has been thought to be caused by increased hepatic BA synthesis (marked by a higher level of serum BA synthetic intermediate 7α-hydroxy-4-cholesten-3-one) [13–15]. We further uncovered that the *Clostridia*-rich gut microbiota also contribute to intraluminal BA metabolic disturbance and thus suppress intestinal BA feedback control of BA synthesis, leading to diarrhea [16]. Hence, the dysregulated gut microbiota–host BA metabolism is a critical promotor of IBS-D. Given that the BA pool has been reported to be changed in specific TCM patterns of GI diseases [17–19], we suppose that the BA metabolic phenotype should be altered with the LDSD pattern of IBS-D.

This study enrolled 221 IBS-D patients with the LDSD or non-LDSD pattern and 80 matched healthy controls. Serum BA indices, fecal BA, and fecal microbiota profiles were individually tested to obtain LDSD-related BA metabolic phenotype.

## Materials and methods

### Subject recruitment and pattern differentiation

Adults (18–65 years) meeting the Rome IV criteria for IBS-D [20, 21] were recruited at Chinese medicine clinics affiliated with the School of Chinese Medicine, Hong Kong Baptist University. By referring to the TCM pattern differentiation criteria previously published [22], each patient was grouped into LDSD or non-LDSD (including spleen-stomach heat-dampness, spleen-kidney yang deficiency and spleen deficiency with dampness) based on the phenotypes of tongue coating, and the conditions of defecation, fatigue, cold-fearing and emotion. Additional age-and sex-matched healthy volunteers without gastrointestinal diseases, BA-related diseases (e.g., metabolic diseases, cardiovascular diseases, psychiatric illness and neurodegenerative diseases) and no surgical histories of gallbladder removal, GI tract, and cerebral cranium were recruited. This study was approved by the Ethics Committee on the Use of Human & Animal Subjects in Teaching & Research of HKBU (Approval no. HASC/15-16/0300 and HASC/16-17/0027), and all recruits signed the written consent form.

Participants meeting the above recruitment criteria were required to complete questionnaires related to IBS-SSS, daily defecation frequency, Bristol Stool Scale, Self-Rating Depression Scale (SDS) and Self-Rating Anxiety Scale (SAS) when the interview with physicians before sampling. The IBS-SSS score was obtained from a 5-item questionnaire measuring the frequency and intensity of abdominal pain, the severity of abdominal distension, dissatisfaction with bowel habits, and the interference of IBS with daily life [23]. The 20-item questionnaires of SAS or SDS developed by Zung et al. [24, 25] were used to evaluate the severity of anxiety or depression, in which the SAS score cut-off value is 50 and the SDS score cut-off value is 53.

Each participant was instructed to provide fasting blood samples and morning first feces once (between 6:00 a.m. and 9 a.m.), required to stop using antibiotics, probiotics, prebiotics and other microbiota-related supplements at least 1 month before sampling. In order to minimize the extra- and intra-individual variation in the stool sampling and BA quantification, all participants were asked to collect the mid-section part of the stool, and each stool sample was fully stirred by technicians before preparing BA extracts.

### Determination of serum BA-related indices

Serum total BAs and fibroblast growth factor 19 (FGF19) were determined by commercial Assay Kit (Thermo scientific, Waltham, MA, USA). Serum 7α-hydroxy-4-cholesten-3-one (C4) was tested based on the liquid chromatography coupled with mass spectrum (LC/MS) as our previously described [16].

### LC/MS-based quantification of fecal BA metabolites

A total of 36 BA standards were purchased from Sigma-Aldrich (St. Louis, MO, USA) and Santa Cruz Biotechnology (Santa Cruz, CA, USA). The isotopic BA deoxycholic acid-2,2,4,4-d4 (DCA-d4) was used as internal standard and was obtained from CDN isotopes (Pointe-Claire, Quebec, Canada). HPLC grade organic reagents for mass spectrometric analysis were purchased from Sigma-Aldrich (St. Louis, MO, USA). BA standards were dissolved in methanol as a stock solution with a concentration of 5 mg/ml. A mixed stock solution was prepared by mixing individual standard stock solution. The standard curves and regression coefficients were gained based on IS adjustment. The signals of each BA metabolites were found in individual measured ranges.

As previously described [16], the fecal sample weighted 100 mg was homogenized with tenfold volume of cold water and then extracted again with another tenfold volume of cold methanol. Supernatants from the two extracts were combined and centrifugated for further BA quantification analysis. fecal BAs were quantified by an LC/MS analytic platform (Agilent UHPLC 1290 and Agilent QQQ-MS 6438, USA) with a single 26-min acquisition with positive/negative ion switching method under multiple reaction monitoring mode. Sample injection and flow rate were set at 2 μl and 0.35 ml/min for each sample, respectively. Bile acid metabolites were separated using an ACQUITY BEH C18 column (1.7 μm, 100 mm × 2.1 mm) with a linear gradient of 0.1% formic acid (FA) in water (A) and 0.1% FA in acetonitrile (B). The gradient program was: 25% to 40% B for the first 6 min, 40% to 70% B for 14 min, 70% to 100% B for 0.1 min, held at 100% B for 2.9 min, then re-equilibration at 25% B for 0.1 min, and held at 25% B for 2.9 min. The column temperature was maintained at 45 °C. The capillary voltage of mass spectrometer was 3.5 kV and 4 kV in positive and negative modes. The acquisition data was analyzed using Agilent MassHunter Workstation Software for peak integration, calibration equations and quantification of individual BAs. Fecal total BA levels of included subjects were obtained by accumulation of all testing BAs.

### Fecal metagenomic sequencing

Microbial DNA was extracted from morning stools (200 mg) with the QIAamp DNA Stool Mini Kit (Qiagen, Hilden, Germany) according to the manufactures’ instructions. The DNA library was established using a TruSeq DNA HT Sample Prep Kit and sequenced by the Illumina Hiseq 2000 at BGI-Shenzhen. By removing low quality bases and human genome, the high-quality sequences were mapped using the published gene catalog database of the human gut microbiome [26]. Microbial diversity was determined, and taxa were identified as described previously [27, 28]. Functional orthologs (KOs) were predicted against the KEGG gene database (v79) by BLASTP with the highest scoring annotated hits. The relative abundances of phyla, genera, species and KOs were calculated from the relative abundances of their respective genes.

### Statistical analysis

Variations of clinical characteristics, bowel symptom severity scores, BA-related indices, fecal BA metabolites were analyzed by the nonparametric Kruskal–Wallis tests for comparison of multiple groups while the Mann–Whitney test was employed for comparison of two groups. Differential taxa and BA-transforming genomes were obtained from the Benjamin–Hochberg method with the *p* value less than 0.1. Relationships of bowel symptom scores with fecal total BA levels or BA-metabolizing species abundances were analyzed by Spearman’s correlation based on Prism 9 (GraphPad Software, La Jolla, CA). The significance difference for all of tests except for fecal metagenomic data was set to *p* < 0.05.

## Results

### A higher incidence of BA overexcretion with more severity of bowel symptoms in the LDSD pattern

The baseline characteristics were summarized in Table 1. There was no difference in the sex ratio and the distribution of BMI, IBS-SSS, SDS and SAS between the LDSD and non-LDSD groups except for the indices of age and fecal BA excretion (*p* < 0.05 resulted from the Chi-square test). There was a lower proportion of elderly patients over 50 years in the LDSD group (19% vs. 34% non-LDSD). By referring to the 90th percentile of fecal total BA levels from healthy subjects [16], a higher incidence of BA overexcretion (≥ 10.61 μmol/g) was shown in patients with LDSD (41% vs. 23% non-LDSD).

**Table 1:** The baseline characteristics of included IBS-D patients with LDSD or non-LDSD pattern

Characteristics	LDSD (n = 105)	Non-LDSD (n = 116)	Chi-square value	*p-*value
Age, n (%)			6.808	0.033
< 30	13 (12%)	16 (14%)		
30–50	72 (69%)	61 (52%)		
> 50	20 (19%)	39 (34%)		
Male, n (%)	54 (51%)	52 (45%)	0.962	0.327
BMI, n (%)				
< 25 kg/m^2^	86 (82%)	93 (80%)	0.171	0.918
25–29.9 kg/m^2^	16 (15%)	20 (17%)		
≥ 30 kg/m^2^	3 (3%)	3 (3%)		
IBS-SSS, n (%)			1.175	0.556
< 175	12 (11%)	14 (12%)		
175–300	57 (54%)	70 (61%)		
> 300	36 (35%)	32 (27%)		
SDS ≥ 53, n (%)	36 (34%)	32 (27%)	1.161	0.281
SAS ≥ 50, n (%)	38 (36%)	39 (34%)	0.160	0.689
BA excretion, n (%) ≥ 10.61 μmol/g	43 (41%)	27 (23%)	7.341	0.007

**Fig. 1:**
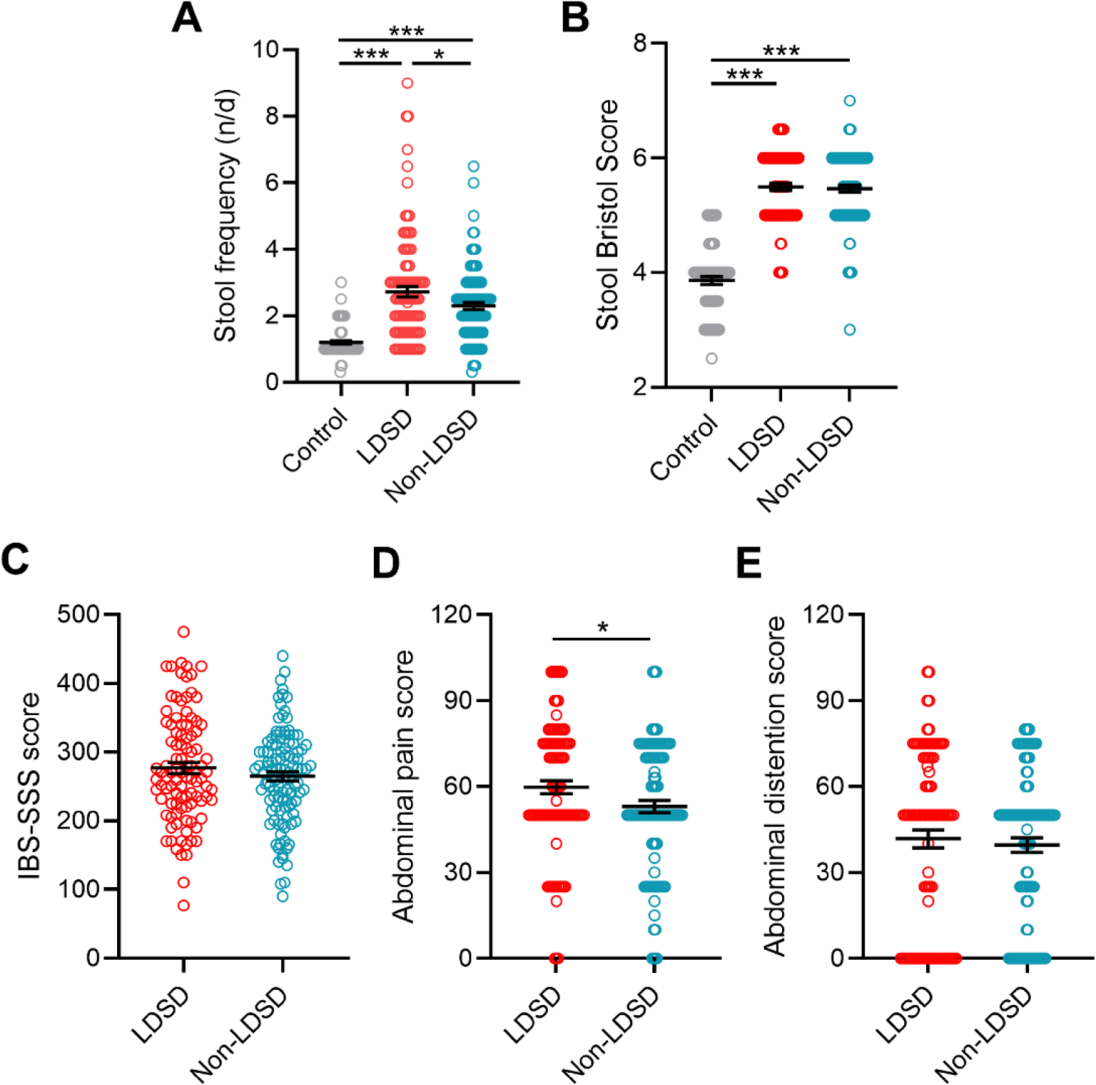
The severity scores of bowel symptoms in IBS-D patients with LDSD or non-LDSD pattern. **A**, **B** The levels of stool frequency and stool Bristol score; **C**–**E** The levels of IBS-SSS, abdominal pain, and abdominal distention scores. Statistical results were obtained from one-way ANOVA with the three-group comparison and unpaired t-test with the two-group comparison (*p < 0.05; ***p < 0.005) and a significant correlation was set as *p* < 0.05

Phenotypic data showed that patients from either LDSD or non-LDSD group exhibited diarrheal symptoms, marked with increased levels of stool frequency and stool Bristol score relative to controls (Fig. 1A, B). Although there was no difference in the levels of stool Bristol score, IBS-SSS score, and abdominal distention score between the two IBS-D subgroups, the LDSD pattern had more frequent daily defecation and a higher abdominal pain score (Fig. 1C–E). As shown above, the LDSD pattern exhibits more severe stool frequency, abdominal pain, and a higher incidence of BA overexcretion.

### An increase of serum C4 with altered fecal BA profile in the LDSD pattern

**Fig. 2:**
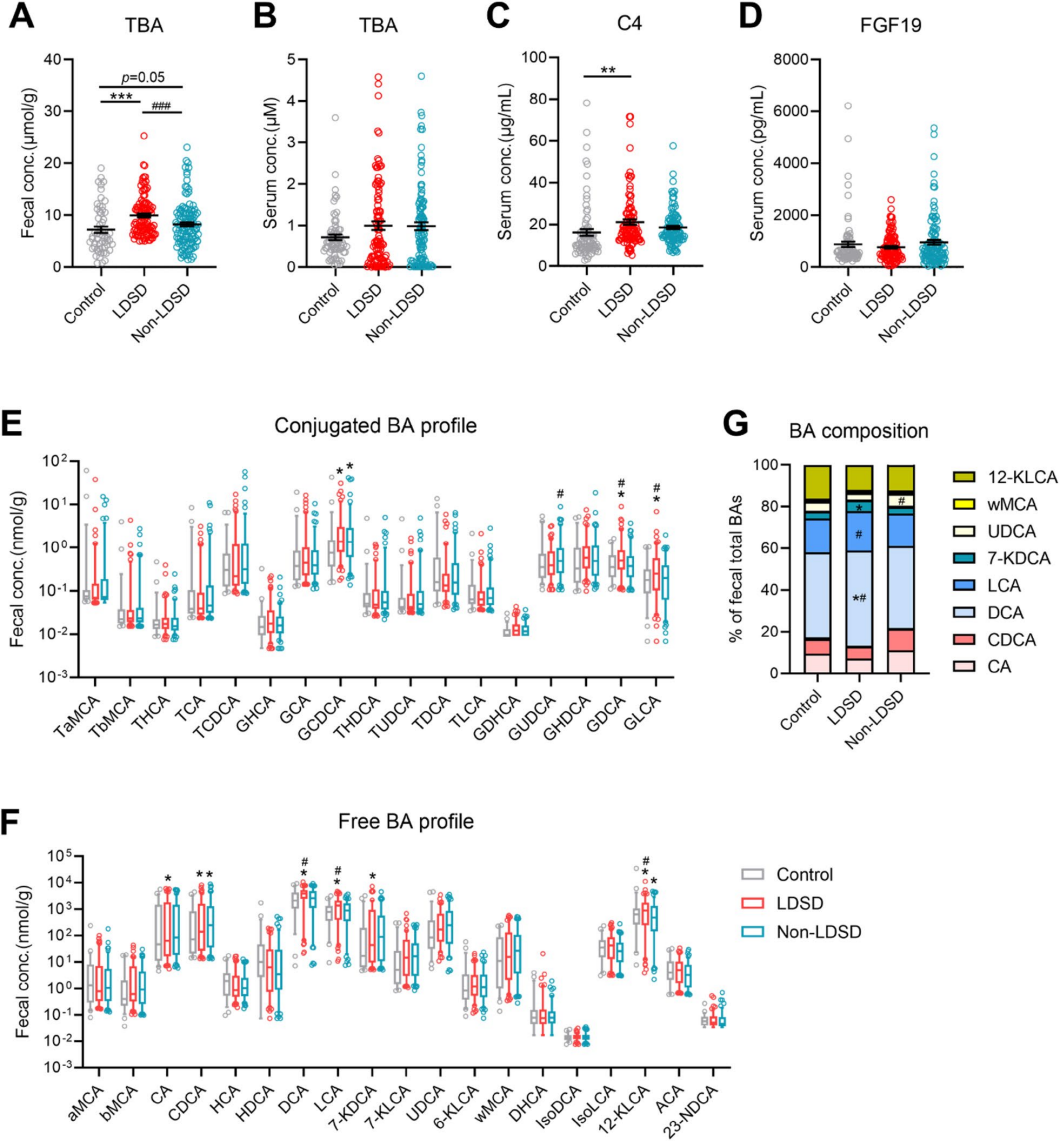
Altered BA metabolic signature of IBS-D patients with LDSD or non-LDSD pattern. **A**, **B** Fecal and serum total BA levels; **C**, **D** Levels of serum 7α-hydroxy-4-cholesten-3-one (C4) and fibroblast growth factor 19 (FGF19); **E**, **F** Fecal concentrations of conjugated and free BA metabolites; **G** The percentages of individual BAs in fecal total BA pool. Statistical data was obtained from one-way ANOVA with the three-group comparison and unpaired t-test with the two-group comparison (*p < 0.05; **p < 0.01; ***p < 0.005 vs. controls; ^#^*p* < 0.05; ^###^*p* < 0.005 vs. the non-LDSD pattern)

In consistent with a higher incidence of BA overexcretion, fecal total BA levels was increased in LDSD patients relative to controls or non-LDSD patients (Fig. 2A). Also, fecal total BA levels were borderline elevated in the non-LDSD group. But serum total BA levels were unchanged in both IBS-D groups (Fig. 2B). Serum C4, a BA precursor as an indicator of BA synthesis [15], was remarkably raised in the LDSD group (Fig. 2C), whereas serum FGF19 released from the intestine with the function to control BA synthesis negatively showed a slight reduction without statistical difference (Fig. 2D).

LC/MS-based BA data revealed that both LDSD and non-LDSD groups showed remarkable increase in levels of primary BA chenodeoxycholic acid (CDCA) and glycochenodeoxycholic acid (GCDCA) in feces (Fig. 2E, F). But secondary BA profile was differed between the two groups. Glycodeoxycholic acid (GDCA), glycolithocholic acid (GLCA), deoxycholic acid (DCA), lithocholic acid (LCA), 7-ketodeoxycholic acid (7-KDCA), and 12-ketolithocholic acid (12-KLCA) were elevated in LDSD patients compared with controls and/or non-LDSD patients (Fig. 2E, F). Fecal BA composition data consistently showed higher proportions of DCA, LCA and 7-KDCA in the LDSD pattern (Fig. 2G). While the non-LDSD group had a higher glycoursodeoxycholic acid (GUDCA) level and an increased proportion of ursodeoxycholic acid (UDCA) (Fig. 2E–G). The above results suggest an enhanced host BA synthesis for the LDSD pattern, with an altered BA-transforming gut microbiome.

### Distinct gut microbiota structures between the LDSD and non-LDSD groups

**Fig. 3:**
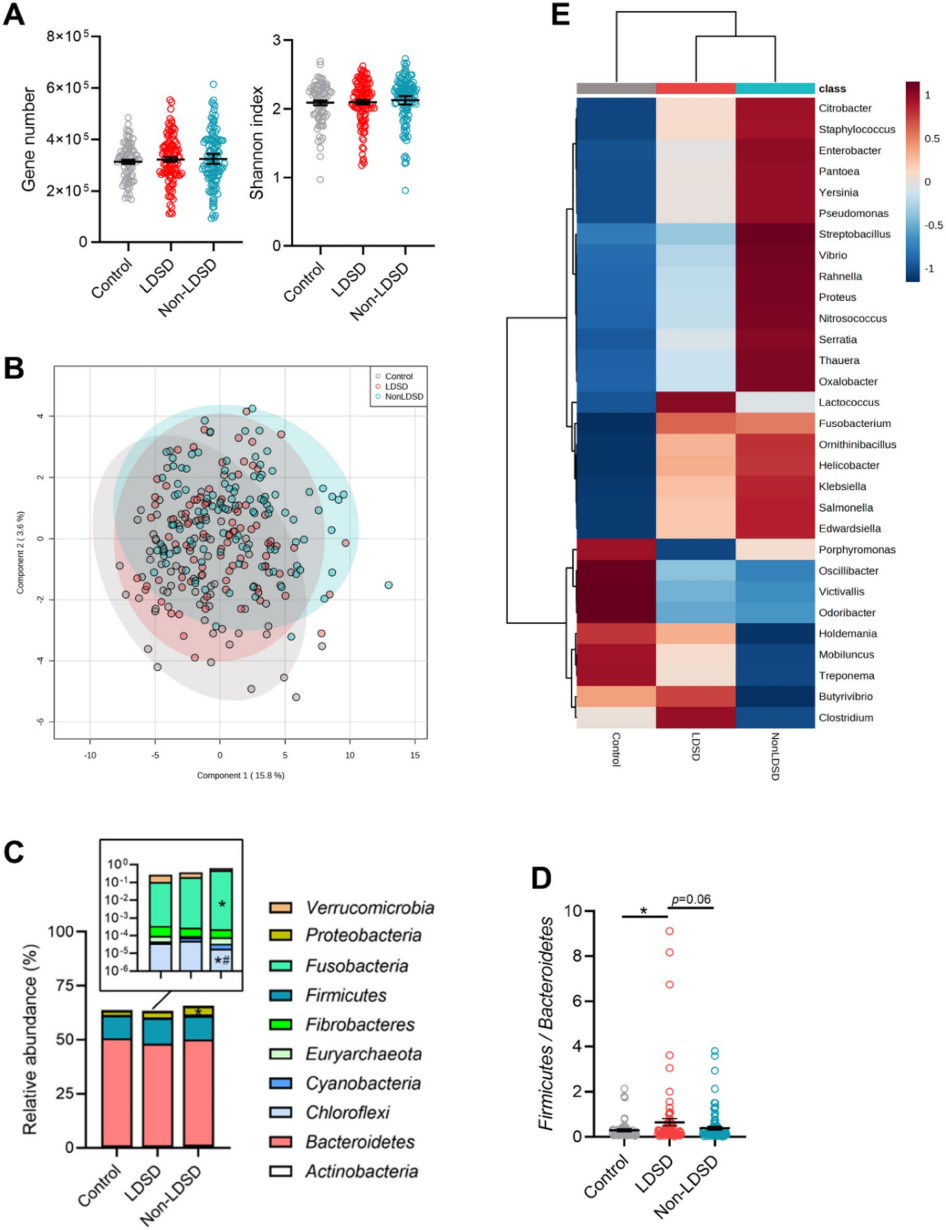
Fecal microbiota signature of IBS-D patients with LDSD or non-LDSD pattern. **A** The levels of gene number and Shannon index; **B** The PCA analysis of fecal microbiota β-diversity; **C** Relative abundances of dominant phyla; **D** The ratio of *Firmicutes* to *Bacteroidetes*; **E** The heatmap of the top 30 differentiated genera. Statistical results were obtained from one-way ANOVA with the three-group comparison (*p < 0.05 vs. controls; ^#^*p* < 0.05 vs. the non-LDSD pattern)

Fecal metagenome data showed that there was no difference in total gene numbers and the Shannon index among the three groups of control, LDSD and non-LDSD, indicating similar gut microbiota α-diversity (Fig. 3A). The PCA analysis also revealed similar microbial β-diversity among groups (Fig. 3B). Still, the taxonomic profile data demonstrated particular differentiation in the fecal microbiota structure among groups. At phylum level, dominant bacteria in the LDSD group did not differ statistically from controls, but the ratio of Firmicutes to Bacteroidetes was increased (Fig. 3C, D). Non-LDSD patients showed increased relative abundances of Proteobacteria and Fusobacteria, while the relative abundance of Chloroflexi decreased (Fig. 3C).

Further, the top 30 differed bacteria (*p* < 0.1 from the Kruskal–Wallis test) was focused to obtain the main change in the taxonomic composition among groups at the genus level (Fig. 3E and Additional file 1: Table S1). The gut microbiota of patients from the two IBS-D subgroups similarly changed, showing that *Bilophila*, *Fusobacterium*, *Helicobacter*, *Ornithinibacillus*, *Ruminococcus*, *Staphylococcus* and *Pantoea* were overabundant while *Victivallis* and *Porphyromonas* were deficient (Fig. 3E and Additional file 1: Table S1). Certain bacteria altered differentially between the two groups, in which genera from *Proteobacteria* (e.g., *Citrobacter*, *Enterobacter*, *Yersinia*, *Pseudomonas*, *Vibrio*, *Rahnella*, *Serratia, Thauera*) and *Fusobacteria* (*Fusobacterium* and *Streptobacillus*) were highly enriched in the non-LDSD group. *Holdemania*, *Mobiluncus* and *Treponema* reversely showed reduced relative abundances (Fig. 3E and Additional file 1: Table S1). Notably, the LDSD pattern had an increased level of *Clostridium* and decreased levels of *Odoribacter* and *Prevotella* (Fig. 3E and Additional file 1: Table S1). Sequence data demonstrated differed gut microbiota signatures between patterns.

### LDSD microbiota featured by altered BA deconjugation and 7-dehydroxylation levels

**Fig. 4:**
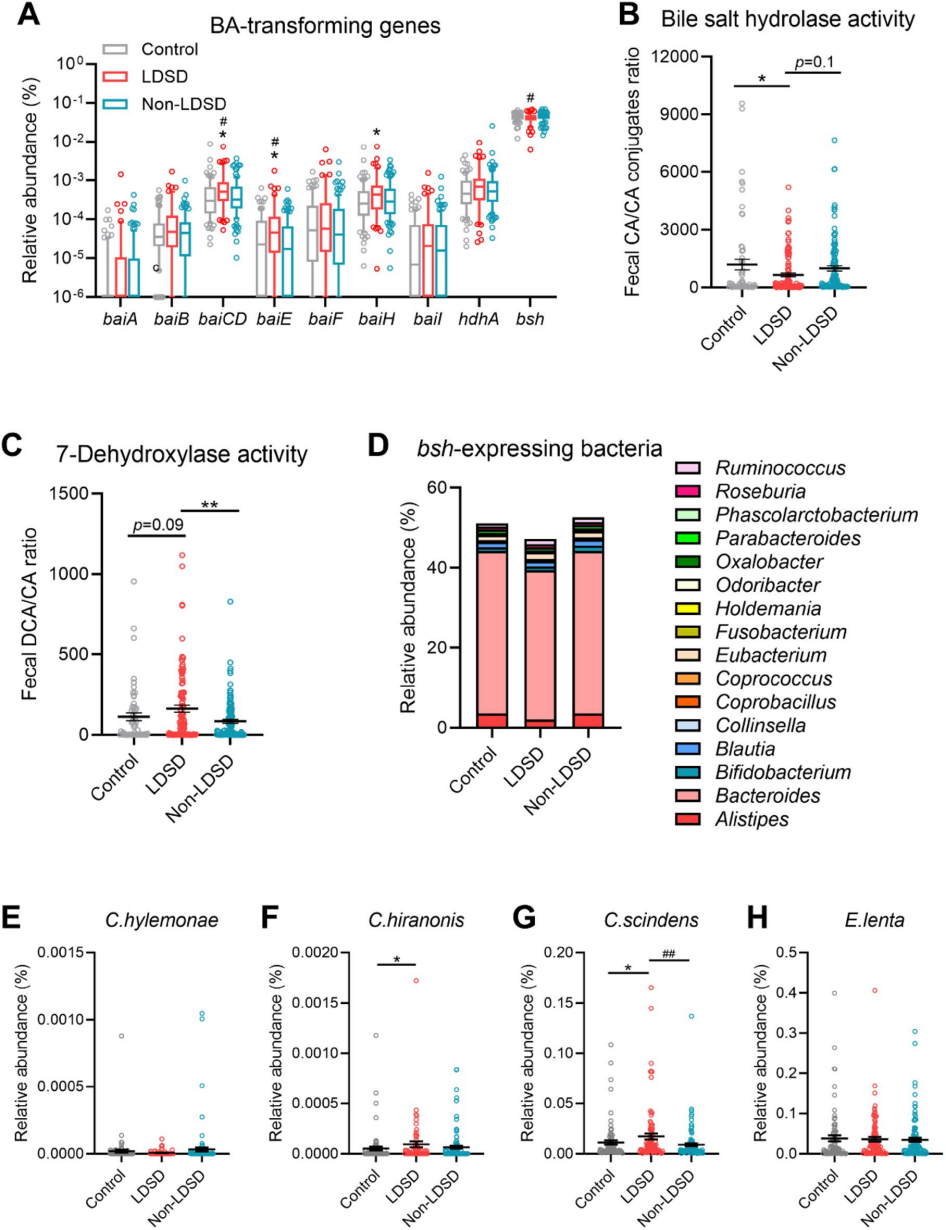
BA-transforming metagenomic signature of IBS-D patients with LDSD or non-LDSD pattern. **A** Relative abundances of 9 BA-transforming genes; **B**, **C** Functional estimation of bacterial bile salt hydrolase and 7-de hydroxylase activities based on the ratios of products to substrates of the two BA-transforming reactions; **D** Relative abundances of *bsh*-expressing genera; **E**–**H** Relative abundances of *bai-*expressing species. Statistical results were obtained from one-way ANOVA with the three-group comparison and Mann–Whitney test with the two-group comparison (*p < 0.05 vs. controls; ^#^*p* < 0.05; ^##^*p* < 0.01 vs. the non-LDSD pattern)

A total of 9 BA-metabolizing genes were identified from the metagenome dataset. Three bile acid-inducible (*bai*) genes, including *baiCD*, *baiE* and *baiH*, showed increased relative abundances in the LDSD group (Fig. 4A). Such three *bai* genes highly dominated in the LDSD pattern is known to encode 7-dehydroxylases that derives secondary BA (such as DCA and LCA) from primary BA (such as CA and CDCA) [29]. Moreover, the gene *bsh*, encoding bile salt hydrolase to deconjugate glycine or taurine from bile salts, had a lower relative abundance. Such genomic results were supported by fecal BA data, a lower ratio of CA to CA amino acid-conjugates and a higher ratio of DCA to CA shown in the LDSD group suggests the LDSD microbiota exerts a reduced bile salt hydrolase activity and an elevated 7-dehydroxylase activity (Fig. 4B, C).

By searching the open-access database VIRTUAL METABOLIC HUMAN (https://vmh.life/), accumulated abundances of *bsh*-expressing genera from Bacteroidetes, Firmicutes, Fusobacteria, Proteobacteria and Actinobacteria was lower in the LDSD group (Fig. 4D). The *bai* genes are expressed in a few species, including *Clostridium hylemonae* (*C. hylemonae*), *Clostridium hiranonis* (*C. hiranonis*), *Clostridium scindens* (*C. scindens*) and *Eggerthella lenta* (*E. lenta*). Among them, *C. hiranonis* and *C. scindens* showed increased relative abundances in the LDSD pattern (Fig. 4E–H). The LDSD gut microbiota enriched by 7-dehydroxylating *Clostridium* species and the *bai* genomes was in accordance with higher levels of DCA, LCA and their glycine conjugations shown in fecal BA profile data.

### LDSD-related BA metabolic changes linked with the severity of bowel symptoms

**Fig. 5:**
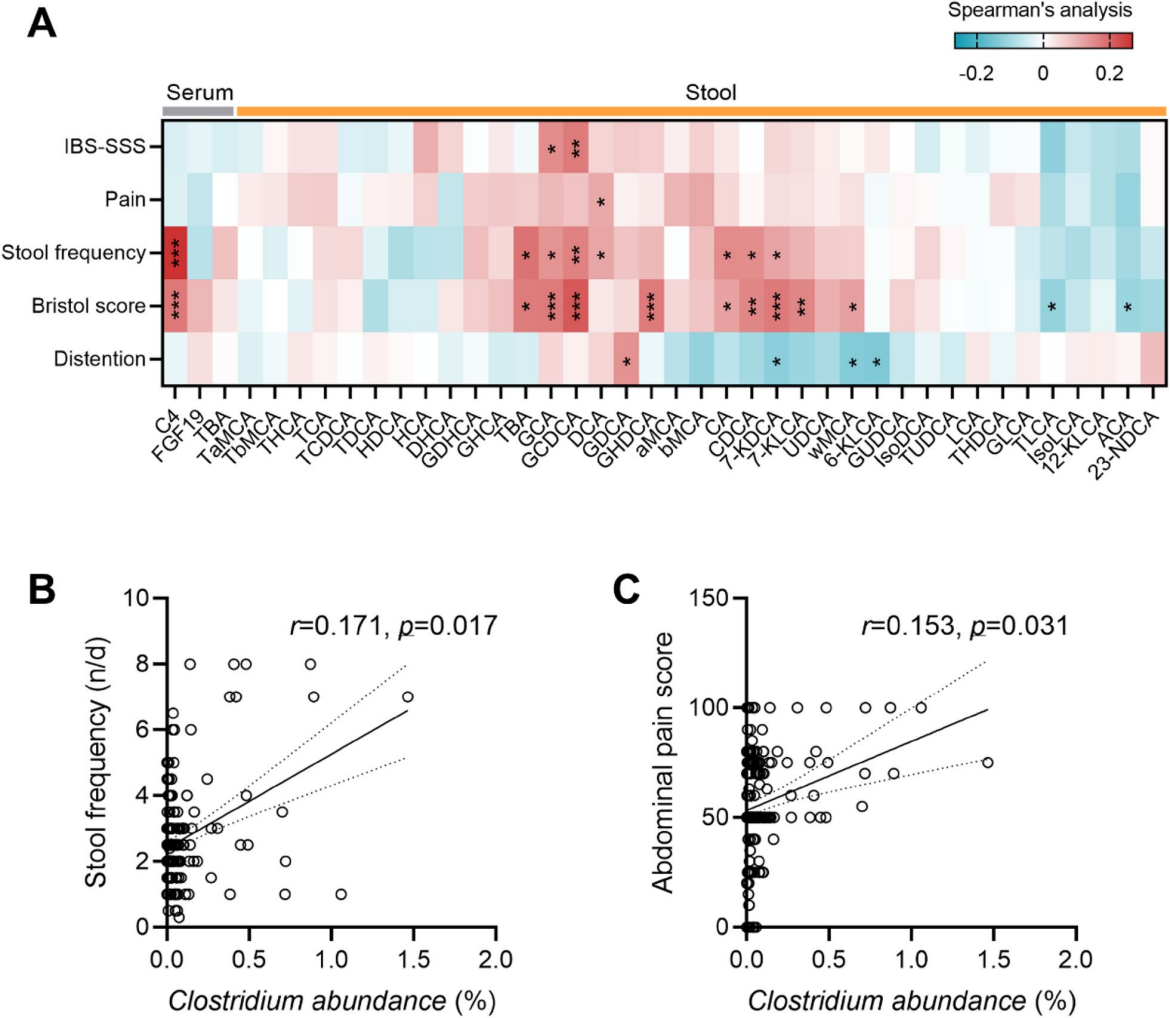
The severities of IBS bowel symptoms strongly correlated with the levels of serum/fecal BA indices (**A**) and *bai-*expressing *Clostridium* species (**B**, **C**) based on the Spearman’s correlation analysis. Significant correlation was set as *p < 0.05; **p < 0.01; ***p < 0.005

We further explored the relationships between the BA metabolic phenotype and bowel symptom severity. Spearman’s correlation data showed that the levels of stool frequency and stool Bristol score were positively associated with the amounts of serum C4 and fecal total BAs (Fig. 5A). Primary BAs (GCA, GCDCA, CA and CDCA) had positive relationships with stool frequency and stool Bristol scores. DCA, a secondary BA derived from microbiota 7-dehydroxylation, showed positive relationships with levels of stool frequency and abdominal pain score (Fig. 5A). Similarly, the relative abundance of *bai*-expressing *Clostridium* species (accumulated by *C. scindens* and *C. hiranonis*) was positively associated with levels of stool frequency and abdominal pain score (Fig. 5B, C). The data demonstrated a particular linkage between LDSD-related BA metabolic phenotype and the severity of stool frequency and abdominal pain.

## Discussion

BA is known to be synthesized in the liver from cholesterol and stored in the gallbladder, secreted into the small intestine when intaking diets, almost reabsorbed from the distal ileum into the portal vein and returned back to the liver; remaining non-reabsorbed BAs are entered into the large intestine and transformed by gut microbiota, finally excreted out of the body in feces [30]. BAs are lost in feces daily, equal to the amount of hepatic synthesis [31]. In terms of the BA metabolic process, the BA overexcretion of IBS-D has been thought to be caused by dysregulated feedback control of BA synthesis, varied genotypes of BA metabolic regulators, increased GI transit, or combination of above factors [32–35].

This study revealed the linkage between the TCM pattern and the BA metabolism in IBS-D for the first time, suggesting that fecal BA overexcretion is associated with more than one TCM pattern of IBS-D, with a closer relation with LDSD. Our data that an increase in the serum C4 level indicate that an excess of hepatic BA synthesis contributes to the BA overexcretion in the LDSD pattern. A slight decrease without significant difference in the serum FGF19 of LDSD patients suggest that the BA overexcretion of LDSD might be independent with intestinal feedback signaling. A recent study reported differential plasma metabolites related to the primary BA biosynthesis in a chronic stress-induced LDSD rats with liver cancer [36], suggesting hepatic BA synthesis may change following LDSD. Another work with the same rat model revealed a tendency of increased gene expression of hepatic BA synthase Cyp7b1 [37]. These findings draw a hypothesis that LDSD could elevate hepatic BA synthetase expression and thus lead to an enhanced BA biosynthetic level, which mechanism deserve to be further investigated. We also noted that the LDSD pattern had a higher level of stool frequency with increased amounts and proportions of fecal free secondary BAs, suggesting that a fast colonic transit might be another contributor of the enhanced BA synthesis and excretion. A positive relationship between the stool frequency level and the amount of serum C4 also support the notion. Above evidence suggests that host BA metabolism of the LDSD pattern is featured with a higher hepatic synthetic level.

LDSD-related gut microbiota was shown to exhibit alteration of BA metabolic function with a reduced level of taurine/glycine conjugation (a lower *bsh* gene level) and a higher level of 7-dehydroxylation (overabundances of *bai* genes and *bai*-expressing *Clostridium* bacteria). The genomic change is indirectly supported by the fecal ratios of products to substrates for the two bacterial BA transforming reactions, although our work had a study limitation in that the comparison of the in vitro BA-metabolizing activity of human fecal microbiota between IBS groups is lacking. Such microbiota BA metabolic change is partially similar to the *Clostridia*-rich microbiota previously described in an IBS-D cohort [16], excluding the change in the *hdhA* gene. The *hdhA* gene with the responsibility of 7-alpha hydroxysteroid dehydrogenase (7α-HSDH) coding is mainly expressed in *Bacteroides*, *Fusobacterium*, *Escherichia*, *Clostridium* and *Ruminococcus* [38]. Of them, *Fusobacterium*, *Ruminococcus* and *Escherichia*, pathobionts that have been reported to trigger GI dysfunction, inflammation and adenoma-carcinoma progression [39–41], were more enriched in the non-LDSD group. It is consistent with an increased levels of GUDCA and UDCA, derived from microbiota 7-HSDH action, shown in the fecal BA profile of the non-LDSD pattern. Omics data indicate that gut microbiota BA metabolism should be varied among TCM patterns.

A study with quantifying LDSD has revealed mental phenotypes closely linked with the liver stagnation factor and abdominal discomfort phenotypes highly related to the spleen deficiency factor [42]. The symptoms of stagnation of liver qi have been closely linked with deficiency of bile secretion and intestinal malabsorption in the field of psychological diseases [43]. Regulator genes involving in BA synthesis (Hnf4a and CYPs) showed elevated expressions in the liver tissue of LDSD rats [37]. Differential metabolic pathways, such as BA biosynthesis, were also shown to closely related to the liver cancer with LDSD in rats [36]. Hence, the enhanced host BA synthesis caused by problemed intestinal feedback control or fast GI transit shown in the LDSD pattern seems to be closely linked with the liver stagnation factor.

Overabundances of *Clostridiales* or *Clostridium* spp. have been reported in the gut microbiota of subjects with spleen-deficient pattern and stress-induced spleen-deficient rodents [44–46]. Reversely, CHM with the spleen-strengthening property (e.g., Qiweibaizhu Decoction) attenuated relative abundance of the *Clostridium* XlVa group [47]. Our data that the increase of *Clostridium* and its produced DCA was found to be positively correlated with the severity of stool frequency and abdominal pain suggest that alterations in the gut microbiota and its BA metabolism are more likely linked with the spleen deficiency factor.

LDSD-related BA metabolic phenotype, particularly the increase of *Clostridium* and its produced DCA, showed positive relationships with the severity of diarrhea and abdominal pain, suggesting a promotive role of gut microbiota BA dysmetabolism in IBS-D progression. Increased colonic BA exposure can induce stool volume and stool habits, particularly the dihydroxy-BAs, such as DCA and CDCA, stimulate water secretion and accelerates colonic transit, increases stool frequency and decreases stool consistency [48, 49]. The underlying mechanism is involved in the BA-driven high-amplitude colonic contractions and increased water and electrolyte secretion. Colonic BA overexposure also promote visceral hypersensitivity through inducing mucosal mast cell-to-nociceptor signaling that involves the farnesoid X receptor/nerve growth factor/transient receptor potential vanilloid 1 axis [50]. DCA, the potent agonist of BA receptor TGR5, could activate subsets of colonic sensory neurons and evoked colonic afferent, thus inducing pronounced visceral hypersensitivity, in a transient receptor potential ankyrin 1 (TRPA1)-dependent mechanism [51]. The BA metabolic phenotype, at least the part shaped by the gut microbiota, is an ideal path to understand the internal relationship between the LDSD pattern and IBS-D progression.

## Conclusion

We reported a featured BA metabolic phenotype of IBS patients with LDSD, characterizing enhanced synthesis and excretion levels for the host and altered BA-transforming genome levels for the gut microbiota. This study targeting the BA metabolism provides foundation toward understanding the biological nature of the LDSD pattern and the internal linkage between the LDSD pattern and IBS progression.

## Supplementary Information

**Additional file 1: Table S1.** Taxonomic difference among groups at phylum and genus levels.

## Data Availability

Data will be made available on request.

## Abbreviations

TCM: Traditional Chinese Medicine
LDSD: Liver depression and spleen deficiency
BA: Bile acid
IBS-SSS: Irritable bowel severity scoring system
SDS: Self-Rating Depression Scale
SAS: Self-Rating Anxiety Scale
IBS: Irritable bowel syndrome
IBS-D: Diarrhea-predominant IBS
IBS-C: Constipation-predominant IBS
IBS-M: Mixed IBS
IBS-U: Un-subtyped IBS
FGIDs: Functional gastrointestinal disorders
LC/MS: Liquid chromatography coupled with mass spectrum
FGF19: Fibroblast growth factor 19
DCA: Deoxycholic acid
LCA: Lithocholic acid
CDCA: Chenodeoxycholic acid
GCDCA: Glycochenodeoxycholic acid
GDCA: Glycodeoxycholic acid
GLCA: Glycolithocholic acid
7-KDCA: 7-Ketodeoxycholic acid
12-KLCA: 12-Ketolithocholic acid
GUDCA: Glycoursodeoxycholic acid
UDCA: Ursodeoxycholic acid
CA: Cholic acid
GCA: Glycocholic acid
GCDCA: Glycocheno-deoxycholic acid
7-HSDH: 7-Hydroxysteroid dehydrogenase
TRPA1: Transient receptor potential ankyrin 1
TGR5: Takeda G protein-coupled receptor 5
